# A Scoping Review of the Use of Non‐Fatty Acid Nutraceuticals for Chronic Inflammation in Persons with Alzheimer’s Disease

**DOI:** 10.1002/alz.090047

**Published:** 2025-01-09

**Authors:** Saajan Patel, Mahi Basra, Kriya Shah, Joshua Caballero

**Affiliations:** ^1^ Nova Southeastern University, Dr. Kiran C. Patel College of Osteopathic Medicine ‐ TBR, Clearwater, FL USA; ^2^ Nova Southeastern Univeristy, Kiran C. Patel College of Osteopathic Medicine, Clearwater, FL USA; ^3^ Nova Southeastern University, Kiran C. Patel College of Osteopathic Medicine, Davie, FL USA; ^4^ University of Georgia, College of Pharmacy, Athens, GA USA

## Abstract

**Background:**

Reducing chronic inflammation has been linked to anti‐inflammatory foods and may be implicated in treating Alzheimer’s disease (AD). Plants produce antioxidants, such as phytochemicals, that appear to reduce the incidence of chronic inflammatory diseases. Phytochemicals may include active substances like polyphenols. Nutraceutical formulations may offer the advantage of combining specific types of phytochemicals with other nutrients to provide optimal efficacy while reducing adverse effects from consuming individual phytochemicals. The purpose of this scoping review was to assess the use of non‐fatty acid nutraceuticals on persons with AD.

**Method:**

A literature search was performed using the databases PubMed, EMBASE and Medline between January 2015 and November 2023. Randomized controlled trials, correlational studies, clinical trials, and case studies were considered, while meta‐analyses, systematic reviews, scoping reviews, animal studies, and non‐English literature were excluded. The search strategy involved key terms such as “Alzheimer”, “Alzheimer’s Disease”, “AD”, “neuroinflammation”, “anti‐inflammatory diet”, “fiber”, “plant extract”, and “supplement”. The PRISMA guidelines guided the inclusion/exclusion process.

**Result:**

After applying the inclusion/exclusion criteria, four out of 49 articles were identified and evaluated. Among nutraceuticals, selenium, carotenoid, combined metabolic activators, and boswellic acid have been studied in randomized controlled trials. Data using selenium (average dose: 0.32 mg tid) over 24 weeks increased selenate cerebrospinal concentrations but did not result in significant cognitive differences compared to placebo. A six‐month study with carotenoid supplementation showed 1‐palmitoyl‐2(5'‐oxo‐valeryl)‐sn‐glycero‐3‐phosphocholine (POVPC) levels, an oxidation marker, did not improve compared to placebo in patients with AD. A study using boswellic showed patients with AD had lower levels of plasma Aβ_40_ and suggested improved MMSE scores. Another study evaluating combined metabolic activators (CMAs) in AD patients showed the administration of CMAs suggested improvement in cognitive function. Limitations among the studies may include patient selection, short duration of trial, determining clinical significance, and assessing costs associated with using these products.

**Conclusion:**

Overall, small, limited studies suggest mild improvements in cognitive function using non‐fatty acid nutraceuticals in persons with AD. Boswellic acid and CMAs may show the most promise. However, larger trials are warranted to confirm preliminary results.

